# Psychological Interventions and Those With Elements of Positive Psychology for Child and Youth Mental Health During the COVID-19 Pandemic: Literature Review, Lessons Learned, and Areas for Future Knowledge Dissemination

**DOI:** 10.2196/59171

**Published:** 2024-09-13

**Authors:** Lynnette Lyzwinski, Jennifer D Zwicker, Sheila Mcdonald, Suzanne Tough

**Affiliations:** 1 Department of Pediatrics, Cumming School of Medicine University of Calgary Calgary, AB Canada; 2 School of Public Policy University of Calgary Calgary, AB Canada; 3 Department of Community Health Sciences University of Calgary Calgary, AB Canada

**Keywords:** positive psychology, mindfulness, resilience, mental health, flourishing, knowledge translation, depression, anxiety, stress

## Abstract

**Background:**

There was a marked decline in child and teenage mental health worldwide during the pandemic, with increasing prevalence of depression, anxiety, and suicide. Research indicates that positive psychological interventions may be beneficial for mental health.

**Objective:**

The aims of this review were to evaluate positive psychological interventions for child and youth mental health implemented during the COVID-19 pandemic and assess overall effectiveness for mental health and knowledge.

**Methods:**

We undertook a literature search of PubMed, MEDLINE, and Google Scholar for all eligible studies on digital and hybrid in-person psychological interventions for youth mental health during the COVID-19 pandemic. A particular emphasis was placed on positive psychological interventions or interventions that had components of positive psychology, including gratitude, acceptance, positive emotions, or resilience building.

**Results:**

A total of 41 interventions were included in this review. Most of the interventions were digital. Overall, most of the interventions assisted with one or more mental health or psychological indicators, such as depression, anxiety, posttraumatic stress disorder, stress, and resilience. However, findings were mixed when it came to targeting both depression and anxiety together. The interventions that promoted youth mental health most often had a range of diverse positive psychology components and were evidence based. Not all studies measured changes in mindfulness. Few studies examined knowledge acquired on mental health self-care, managing mental health problems, knowledge of positive psychological techniques, mindfulness knowledge, or mental health self-efficacy.

**Conclusions:**

Diverse multicomponent interventions appear to assist with youth mental health overall, although their effects on both depression and anxiety are less clear. There is also a need for more research on knowledge gains to determine whether the interventions improved knowledge on mental health–supportive behaviors, which may be sustained beyond the intervention. Finally, more studies need to evaluate whether the interventions assisted with increasing self-efficacy for practicing positive psychological techniques as well as changes in mindfulness levels. Future studies should not only assess effectiveness for mental health outcomes but also assess knowledge translation, with valid measures of knowledge and self-efficacy for mental health–supportive behaviors and positive psychological skills acquired (eg, the ability to practice mindfulness).

## Introduction

### Background

Worldwide, there was an increase in mental health problems in children and teenagers, including increasing rates of depression, anxiety, and suicide, during the global COVID-19 pandemic [1]. Systematic reviews have estimated the global prevalence of childhood and teenage depression at 23% and anxiety at 28% [2], whereas the prevalence of suicidal ideation was estimated to be 29% to 33% in this age group [3]. A study in Australia found an increase in the use of helplines for kids to cope with mental health issues [4]. Higher levels of isolation were reported in children and teenagers resulting from the closure of schools and reduced socialization with peers, coupled with challenges stemming from reduced access to mental health services [1,5]. Moreover, data from the World Health Organization (WHO) indicate that there were barriers to accessing mental health care services, particularly among children and teenagers, who experienced a 72% disturbance in their mental health care, with counseling and psychotherapy being the leading services that were affected during the transition to digital care [6].

Given the serious consequences for child and youth mental and psychological health, there is a need to evaluate what strategies were effective for buffering against the deleterious impact of the pandemic. Without understanding what measures were effective for helping children and youth cope and adjust to the “new normal” gaps and barriers will remain when it comes to future pandemic preparedness in relation to mental health crises in this population.

A systematic review comparing the prepandemic and pandemic periods found that youth experienced higher levels of isolation, depression, and anxiety during the pandemic [7]. Data from the WHO suggest that children from economically-deprived backgrounds were impacted the most [8]. Research also suggests that certain personality and neurodevelopmental types were differentially affected during the pandemic. For example, research has found that children with neurodevelopmental disorders had higher anxiety during the pandemic [9] and that siblings often provided social support for distressed children with disabilities [10]. In addition, a systematic review found that some children experienced boredom during the pandemic [11], which was also found in other studies among neurotypical individuals [9,12,13].

The pandemic also created challenges for parents in terms of adjusting to learning from home [14]. Research from the All Our Families cohort in Calgary, Alberta, also found a rise in depression by 3.2 points and in anxiety by 2.39 points in mothers during this period when compared with the 3-year period before the pandemic [15]. They struggled with managing homeschooling, lack of daycare, and financial troubles [15].

Research from the WHO indicates that social support was a major buffer for promoting mental well-being in children and teenagers during the pandemic [8]. The prevailing literature also suggests that self-care, positive psychological states, and resilience have protective effects on well-being, stress management, and mental health [16-23]. Positive psychology is described as a state of flourishing and thriving even when experiencing health problems or hardship by living one’s best life [19]. Individuals who have high levels of positive mental health and resilience [24] and who score high on resilience tend to have lower levels of depression [24] and anxiety [23]. Individuals with high levels of positive mental health are 27 times more likely to recover from mental health issues relative to their counterparts with low levels of positive mental health [25]. Furthermore, positive mental health has been found to be a buffer for suicide prevention in patients with depression [26].

Positive psychology is relevant to children and youth in relation to their mental health during the pandemic. Positive psychology can help youth by providing them with the necessary tools and resources to increase their resilience, well-being, and ability to cope during difficult times, which is especially relevant to the global youth mental health crisis during the pandemic [16-23]. Individuals can be struggling with mental health problems, yet they can demonstrate resilience and flourish. Flourishing focuses on thriving amidst hardship by living one’s best life even when experiencing metal health problems or other difficulties [18,19] and is particularly relevant for youth, given the mental health crisis worldwide [18,19]. Flourishing has several essential elements, which include life satisfaction, meaning or purpose in life, character or virtues, and positive affect. It also includes happiness, social relationships, and mental health [27-29].

Resilience is a form of internal coping in one’s given environment regardless of external stressors that one may encounter [30]. Resilience has also been found to be inversely related to mental health problems and a buffer against stress [21]. Building resilience and helping youth find ways to flourish include assisting youth in acquiring the skills and tools to cope during difficult life circumstances [20]. Considering the relevance of positive psychology and its components for youth mental health, more research is needed to better understand the types of positive psychological interventions that have been developed, including ones that aimed to increase flourishing and build resilience in youth during the COVID-19 pandemic and their overall effectiveness for mental health.

In addition to positive psychology, there are also psychological interventions that are closely connected to it and potentially of great importance for youth mental health during the pandemic. Research indicates that mindfulness, a form of present-moment awareness using formal and informal meditation, may help build resilience through greater self-awareness, acceptance, kindness, and gratitude [31]. Mindfulness also has elements that are of great importance to positive psychology, including self-acceptance, compassion, positive emotions, and gratitude [32,33]. Research also indicates that mindfulness practice may help with the development of compassion and reducing stress, anxiety [34], and depression [10-13]. Studies have further found that, when students are more mindful, they can learn more in the classroom and be more focused on given tasks and are more creative [14,15]. Mindfulness is of relevance to mental well-being in children and youth during the pandemic [34]. Cross-sectional research on mindfulness in children has found that higher levels of mindfulness (children who were more mindful) were linked to higher coping during the pandemic [34]. Even with the pandemic coming to an end, young people may still face hardship and personal crises, and there is a need to provide them with coping skills.

Finally, research has found that higher levels of social support, coupled with positive coping strategies, were protective for youth mental health during the pandemic [35]. Recommendations for helping children and adolescents have included developing coping strategies as well as receiving social support from family members [2]. Thus, investigating interventions that have assisted youth with positive coping and psychological and social support during the pandemic to promote youth mental health can inform strategies for future mitigation of mental health concerns in other crises.

Furthermore, to the best of our knowledge, there has not been a review that has evaluated what types of positive psychological interventions have been undertaken for youth mental health, including their fundamental components and whether they were effective for alleviating mental health and psychological indicators such as resilience, stress, and well-being during times of unprecedented crises, stemming from the pandemic.

In addition to traditional positive psychological interventions, there also has not been a review that has explored psychological interventions that have common elements that are related to traditional positive psychology, such as gratitude and resilience building [32,33]. Thus, it is also of interest to explore interventions that contain elements that are related to positive psychology, such as mindfulness, which focuses on self-acceptance, compassion, and gratitude, among other things [32,33]. Some experts also argue that cognitive behavioral therapy (CBT) has important elements that are of fundamental relevance to positive psychology, including its focus on positive emotions [36], hope, and strength building, highlighting that it is useful to include interventions that are informed by CBT as well. In recent times, there has also been a shift toward a “positive CBT” model of care whereby the core treatment focus is on positive aspects exclusively [37].

In addition, little is known about the uptake of mental health–promoting behaviors, such as mindfulness, from positive psychological interventions. For example, studies have examined whether mindfulness interventions assist with mental and psychological health but have not measured knowledge levels, and few have measured changes in mindfulness [38,39]. There is a need to investigate whether positive psychology interventions help youth with gaining important knowledge surrounding mental health as well as techniques derived from positive psychology, such as mindfulness for self-care. Mental health literacy has also been found to be an important determinant of help-seeking behaviors [40], and exploring it in relation to positive psychological interventions in youth during the pandemic is of research interest. Understanding whether the interventions effectively disseminated knowledge on self-care in relation to mindfulness and knowledge of positive psychological techniques to assist with coping is also important. Often, research is not translated into practice [41], and developing effective knowledge dissemination strategies enables evidence-based research findings to take shape in the community through increased awareness and greater adoption of these mental health–promoting resources and behaviors.

### Objectives

This review had the following aims:

1. To better understand what positive health psychology interventions (including resilience and flourishing focused), as well as interventions that have common elements related to positive psychology (eg, mindfulness-based interventions or CBT-based interventions [36]), were developed during the pandemic and their overall effectiveness for youth mental health, including depression, anxiety, and well-being.
2. To better understand whether the psychological interventions increase levels of mindfulness and resilience in youth.
3. To better understand whether the psychological interventions increase knowledge of positive psychology practices, including knowledge levels of mindfulness in youth and knowledge of mental health self-care behaviors. In other words, did the interventions assist with knowledge translation (KT) on how to practice self-care and resilience coping skills?

## Methods

### Study Design

A literature search was undertaken in PubMed and MEDLINE for all relevant studies on traditional positive psychological interventions or psychological interventions with elements that are common to positive psychology for child and youth mental health during the pandemic, using the PRISMA-P (Preferred Reporting Items for Systematic Reviews and Meta-Analyses for Protocols) guidelines [42]. Google Scholar and manual hand searches were also undertaken to identify any additional studies.

### Inclusion and Exclusion Criteria

All positive psychological interventions studies on children (aged <10 years), preteenagers (aged 10-12 years), teenagers (aged 13-18 years), or youth and student mental health (ages of 18-24 years) were included if they were undertaken during the pandemic period (March 2020 to December 2022). The search was undertaken in December 2022 and recently updated in July 2024 to capture additional studies that were undertaken during the pandemic but published at a later date. Studies that were designed before the pandemic but evaluated the effects of the intervention during a period of the pandemic were also included. We included mindfulness-based interventions, CBT, flourishing, and resilience, among other interventions that fell under the positive psychology umbrella for youth mental health. Reviews were excluded, as well as articles that were not published in peer-reviewed journals. Studies must have been published in the English language to be included. Qualitative studies were excluded. Studies must have been interventional in design, such as randomized controlled trials (RCTs) or quasi-experimental 1-arm pretest-posttest designs. Pilot RCTs were included. Studies on program implementation or health care service delivery experiences or perspectives were excluded. Only studies that evaluated the effectiveness of positive psychological interventions were included.

### Search

A medical librarian assisted with the search strategy. Keywords included word variations for “mindfulness” or “intervention” and “mental health” or “flourishing” or “resilience” or “stress” or “positive psychology” and “young adult” or “teenager” or “youth” and “knowledge” or “knowledge translation.” The population included young adults, children, and teenagers. The interventions included positive psychological interventions, mindfulness-based interventions, and CBT, among others. The outcomes included depression, anxiety, stress, and resilience, among others. The comparator included a control group (standard or usual care), but single-arm pretest-posttest studies did not have a comparator. Word variations for each outcome, intervention, and population were entered into the search. The search was rerun and updated again in July 2024 to ensure that all relevant studies were still included. An example of the search strategy is detailed in Textbox 1.

**PubMed search strategy example.**

**Search strategy**

- (“Adolescent” [Medical Subject Heading (MeSH)] OR teen [ti] OR teens [ti] OR teenage* [ti] OR adolescen* [ti] OR youth [ti] OR youths [ti] OR “young people” [ti] OR “young adult” [ti] OR “young adults” [ti] OR “Child” [MeSH:NoExp] OR child* [ti] OR family [mh] OR caregiver [mh] OR parent [ti] OR parents [ti] OR parental [ti] OR familial [ti] OR family [ti] OR families [ti] OR mother* [ti] OR father* [ti] OR caregiver [ti]) (“mindfulness” [MeSH] OR “mindfulness” [all fields] OR mbct [tiab] OR mbsr [tiab] OR “Mindfulness-Based Cognitive Therapy” [tiab] OR “Mindfulness Based Stress Reduction” [tiab] OR “MBI” [tiab] OR “mindfulness-based interventions” [tiab] OR meditation [tiab] OR “Mental Disorders/therapy” [MAJR] OR ((“Mental Health” [MeSH:NoExp]) AND “First Aid” [MeSH:NoExp]) OR “Psychological First Aid” [MeSH] OR “mental health first aid” OR “psychological first aid” OR “resilience, psychological” [mh] OR “resilience” [tiab] OR “hardiness” [tiab] OR “posttraumatic growth” [tiab] OR “post-traumatic growth”[tiab] OR “personal growth” [tiab] OR “psychological well-being” [tiab] OR “stress related growth” [tiab] OR “coping behavior” [tiab] OR “emotional stress” [tiab] OR “mental health”[MeSH] OR “mental health” [tiab] OR “flourishing” OR “flourish” OR “Emotions” [MeSH] OR “positive psychology” OR “Psychological Recovery”) (“diffusion of innovation” [mh] OR “diffusion of innovat*” [Tiab] OR “information dissemination” [mh] OR “knowledge util*” [tiab] OR “knowledge uptake” [Tiab] OR “knowledge transfer*” [Tiab] OR “knowledge implement*” [Tiab] OR “knowledge disseminat*” [Tiab] OR “knowledge translat*” [Tiab] OR “research utiliz*” [Tiab] OR “research uptake” [Tiab] OR “research transfer*” [Tiab] OR “research implement*” [Tiab] OR “research disseminat*” [Tiab] OR “research translat*” [Tiab] OR “health services research” [tiab] OR (“utili*” [Ti] AND “review” [Ti]) OR implement* OR train*) (“covid 19” [tiab] OR “covid 19” [MeSH] OR “sars cov 2” [tiab] OR “sars cov 2” [MeSH] OR “severe acute respiratory syndrome coronavirus 2” [all fields] OR “ncov” [all fields] OR “2019 ncov” [all fields] OR “Pandemics” [MeSH])

### Screening and Data Extraction

Titles were screened for relevance followed by screening of abstracts against the inclusion and exclusion criteria, followed by retrieval of full-text articles. Full texts meeting the inclusion criteria were included in the review. Data were extracted and summarized in tabular format. This included the study general characteristics, measures, outcomes (mental health), positive psychology intervention types, mindfulness attributes, behavior change techniques, intervention details, and knowledge.

## Results

### Overview

**Figure 1:**
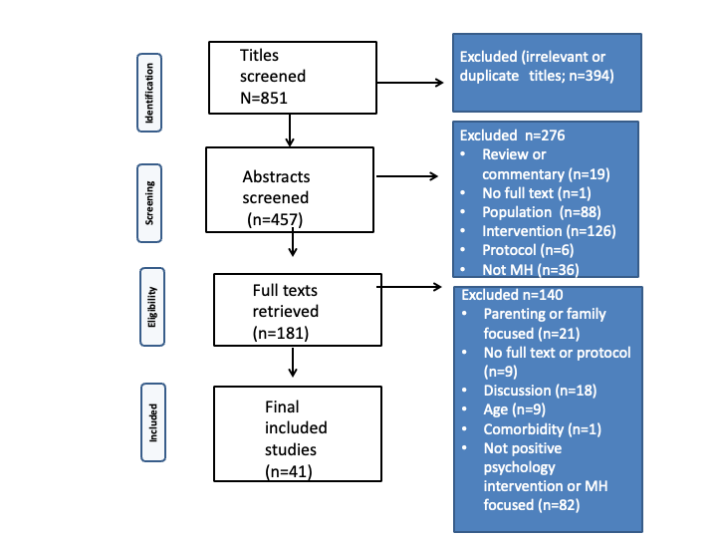
Primsa flow chart.

A total of 41 psychological interventions targeting youth during the pandemic met the inclusion criteria and were included in the review [43-83] that were either purely positive psychological interventions or had common elements that are of great relevance to positive psychology. The results are summarized in Multimedia Appendix 1 [43-55,57-74,76-78,80-83]. Most of the interventions were delivered digitally during the pandemic. The interventions included web-based interventions, mobile health apps, videos, web-based audios and lectures, and emails. In total, 3 were apps, of which 2 were chatbot apps [45,52,60]. A total of 5% (2/41) of the interventions were delivered over WeChat (Tencent) [54,66]. The studies spanned the United States, Canada, Australia, the United Kingdom, Italy, Portugal, Spain, China, Iran, Tunisia, and Vietnam. The search, including the screening process, is illustrated in Figure 1.

### Intervention Content

Almost all of interventions were digital, with the majority being delivered through a web-based mobile health app-based platform [45-47,49-56,58-68,70-73,76,79-82]. Several studies were of mindfulness-based interventions or included mindfulness components [43,44,46,50-52,56,57,60,66-69]. Some of these interventions combined various elements of mindfulness, such as acts of gratitude, breathing, and meditation, whereas 5% (2/12) included mind-body movement with yoga [44,45]. In total, 7% (3/41) of the interventions included more formal mindfulness techniques derived from mindfulness-based stress reduction (MBSR) [43,56,66]. There were also several CBT based studies [54,55,58,59,61-65], as well as mixed hybrid CBD studies involving other techniques such as combined CBT with dialectical behavior therapy (DBT) [65] and combined mindfulness with CBT [62,64]. A few of the studies had a social support component, with one comparing CBT with social support informed by the social identity framework [48], another including social support with DBT and other positive psychology principles [65], and a third including social support with mindfulness [57]. One study had a spiritual element, focusing on prayer with a counselor along with meditation [49]. Additionally, a few of the studies combined several different positive psychological components, such as positivity, compassion, and meaningful engagement [53] or self-care, compassion, and meditation [47].

### Positive Psychological Interventions for Youth Mental Health

#### Overview

Overall, almost all of the psychological interventions targeting youth mental health had positive results on one or more mental health outcomes, but a monitory of the studies did not find that the interventions were effective for any of the measured mental health outcomes [44,50,51,67]. The studies with negative findings often tended to not include a thoroughly described program and theoretical underpinning. For example, they did not describe the details of the mindfulness program [50] or vaguely described informal mindful photography practice with acts of kindness [51] or mind-body movement with breathing [44] rather than relying on evidence-based structured programs with mindfulness techniques that were described in greater detail.

#### Effects on Depression

Many of the studies that measured depression found that the interventions improved depression [43,45,53,54,58,59,61,65,66,68,71,73,74,77,80], but a minority not find that the interventions assisted with depression [62,64]. Of these studies, some were on mindfulness-based interventions [43,66,68], while others were predominantly CBT interventions [54,59,61,65], with one combining resilience with CBT training [61] and another combining CBT with DBT [65]. In addition, one was a multicomponent positive psychological intervention [53], whereas another was a yoga intervention [45]. Social support was found to be superior to CBT in one study for depression [48], whereas another study found that social support was as effective as mindfulness for alleviating depression even though mindfulness was helpful [66]. A web-based intervention involving Ipsha yoga with audios, found that depression was alleviated in the intervention arm but only at week 2 [45].

#### Effects on Anxiety

Several of the studies that measured changes in anxiety found that the positive psychology interventions assisted with anxiety. Of the studies that demonstrated effectiveness, one was a multicomponent positive psychological intervention [53], several were mindfulness-based interventions [43,52,64,66,70,72,82] as well as CBT based [54,55,58,59,63,64,73,74,77].

#### Effects on Both Anxiety and Depression

Although several studies found independent effects of the interventions on improving either depression or anxiety, some studies had mixed findings with regard to the effects of the interventions on effectively targeting both depression and anxiety. In other words, some studies found that the interventions assisted with both depression and anxiety together, whereas others did not. Several studies found that the interventions were effective for decreasing both depression and anxiety [43,53,54,58,59,66,73,74,77]. For example, the 8-week MBSR randomized pilot study in 49 participants by An et al [43] found that the intervention reduced depression and anxiety by 40% and was associated with changes in the brain on electroencephalogram readings. In addition, one study was a multicomponent 8-week positive psychology RCT involving 366 participants and found reductions in depression and anxiety [53].

A few studies did not find that the interventions reduced both anxiety and depression together [45,61,64,70,80,81]. For example, the 4-week MBSR with mindfulness-based cognitive therapy (MBCT) RCT (N=144) by Sun et al [66] found reductions in anxiety but not depression in the intervention arm relative to the control arms. Furthermore, while the 4-week CBT RCT (N=585) by Rackoff et al [61] found that the intervention reduced depression, there were no substantial changes in anxiety. In contrast, the 8-week CBT RCT (N=177) by Simonsson et al [64] found reductions in anxiety but not depression in youth. Both studies had some elements of mindfulness. In addition to this, the 8-week web-based CBT plus mindfulness intervention by Ritvo et al [62] did not have any significant effects on depression or anxiety. This contrasts with a CBT chatbot app that reduced depression and anxiety levels in youth [54].

#### Effects on Posttraumatic Stress Disorder

A total of 4% (2/41) of the studies evaluated the effects of the positive psychology interventions on posttraumatic stress disorder (PTSD) in youth during the pandemic and found significantly lower levels of PTSD in intervention participants relative to controls [57,59].

### Positive Psychological and Derived Interventions for Youth Psychological Stress and Well-Being

Most of the studies that evaluated stress found that stress was reduced in youth after the psychological interventions [43,45,46,49,52,53,61,62,68], but it should be noted that one of these studies found only marginal effects [52]. Well-being was also higher in the intervention arms relative to the control groups in a few studies that assessed it [45,46,53]. Youth also scored higher on various emotional facets and character traits, including positive affect [45], hope, optimism, emotional regulation [53], forgiveness [60], and life satisfaction [60].

### Flourishing and Its Core Elements: Affect, Life Satisfaction, Character, Meaning, and Relationships

We did not find any positive psychology interventions that measured and evaluated human flourishing in youth at baseline and follow-up during the pandemic. However, a minority of the positive psychology interventions included fundamental elements of human flourishing from its core definition [27,29]. This included character, positive emotions, meaning, happiness, purpose, life satisfaction, social relationships, affect, and mental health [28,52,53,61,62].

The study by Krifa et al [53] found that multiple positive psychological interventions may be superior for youth mental health by combining compassion, positivity, and meaningful engagement in a digital intervention. Although they did not directly measure flourishing, these elements are fundamental complements of flourishing [53]. The 2-week RCT (N=250) by Dupont et al [51] focused on small acts of kindness, gratitude, and mindful photography, although the study did not have significant effects on any mental health outcomes of interest. The 2-week RCT (N=164) by Pizarro-Ruiz et al [60] involved an app that promoted positive affect, life satisfaction, and forgiveness. Similarly, the study by Rackoff et al [61] targeted positive affect but also included elements that focused on building healthy relationships and coping skills and finding a greater meaning in life. The 8-week RCT (N=154) by Ritvo et al [62] combined mindfulness with other positive psychological elements linked to flourishing, including life satisfaction, healthy relationships, coping skills, and a healthy lifestyle. Similarly, the 4-week pretest-posttest study by Gabrielli et al [52] (N=71) combined mindfulness with behavioral coping strategies that assisted with emotional regulation. The 4-week RCT (N=190) by Dorais and Gutierrez [49] focused on meaning and purpose in life through prayer.

### Knowledge Uptake

Almost none of the studies evaluated knowledge dissemination and uptake levels for mental health knowledge and self-care knowledge, among other key knowledge-related domains relevant to the positive psychology interventions. Only one of the studies, the study by Sun et al [66], evaluated knowledge in relation to self-care, regulating emotions, the mind and body, and coping with stress. The study by Sun et al [66] found that knowledge of self-care behaviors, emotional regulation, and stress coping improved after the intervention. Although one study found that participants had higher levels of emotional regulation, it did not assess knowledge levels [53]. In addition, only one study evaluated motivation to learn and found that participants were motivated to continue to learn about self-care in a compassion, self-care, and meditative intervention [47].

In addition to knowledge gained and motivation to learn, 4% (2/41) of the studies considered self-efficacy for managing mental health. One study assessed changes in mental health self-efficacy and management but did not find that the intervention increased overall levels [65], whereas another found that mental health self-efficacy increased [58].

Furthermore, none of the studies evaluated self-efficacy to practice mindfulness or other positive psychology techniques, including CBT. Only one study, the study by Sun et al [66], reported that participants had learned how to practice mindfulness, including self-regulation of the mind and body, whereas another study found that participants enjoyed learning the mindfulness modules [52]. This suggests that only a minority of studies considered knowledge and skill acquisition in relation to positive psychological techniques, whereas none considered self-efficacy.

### Study Quality

Overall, the studies were of average quality when considering several factors, including those listed by the Cochrane Risk of Bias grading tool [50]. The study duration ranged from 2 weeks to 1 year. Mindfulness-based interventions are traditionally 8 weeks in duration; hence, studies shorter than this were graded as weak [84]. Most of the studies were RCTs. Blinding was usually not undertaken except in a few studies in which the assessors or participants were blinded, but not both [49,50,60,61]. Most studies had good overall retention levels. Several studies also undertook a priori power calculations, ensuring that they were sufficiently powered with adequate sample sizes to detect a difference if one existed between the intervention and control arms [44,45,49-51,53,54,61,62,66]. In addition, the studies used data collection methods that were valid and reliable, or the questionnaires had been previously used and validated.

## Discussion

### Principal Findings

We aimed to better understand what positive psychology or positive psychology–derived or related interventions (including ones that had common elements from positive psychology) were implemented to address mental health concerns among youth during the pandemic.

First, we aimed to better understand what types of interventions were implemented and the types of media and techniques that were used. Overall, the interventions that targeted more than one element were more likely to have significant effects on one or more mental health outcomes [53]. The interventions were diverse, often combining various facets of positive psychology along with mindfulness or CBT. Among the interventions, almost all had a CBT or mindfulness component (including informal practice or movement). However, only a minority had a formal mindfulness program derived from MBSR or MBCT, with the rest providing fewer details. The studies in this review included combinations of CBT with mindfulness, combined CBT with resilience training, combined either mindfulness or CBT with social support, as well as combined CBT with DBT. In addition, there was a multicomponent + psychology intervention, informal mind-body movement with yoga interventions, and combined positive psychology with informal mindfulness (eg, gratitude and compassion), and 1 (1/41, 2%) was based on meditative counseling with prayer. We also found that some elements of flourishing, namely, life satisfaction; positive emotions; purpose; and certain qualities, including gratitude, were targeted in the studies. However, none of the studies evaluated flourishing as an entire construct, which could be useful in future positive psychological interventions to determine whether they assist with flourishing.

Second, we further aimed to determine whether the interventions were effective for improving mental health. Overall, the interventions demonstrated effectiveness for improving mental health in youth. Almost all of the interventions helped with one or more mental health outcomes, including depression, anxiety, stress, PTSD, and resilience among youth. Many of the mindfulness-based interventions assisted with depression and anxiety. Previous research has found that mindfulness and CBT are effective for anxiety and depression [85-88], confirming our findings. The prevailing literature on positive psychology also indicates that it helps with resilience and improving positive affect [89,90], which was also found in this review.

Furthermore, the studies were heterogenous in terms of program content, with the interventions that implemented comprehensive media with evidence-based MBSR or MBCT usually showing positive effects compared with interventions that did not elaborate on their programs. Thus, it appears that multicomponent interventions that used diverse media and had greater intervention complexity were more likely to have positive results for mental health in general. For example, only the chatbot study showed significant effects compared with the web-based mindfulness one [52,62]. It could perhaps be that mobile apps for mindfulness combined with other positive psychology elements can best support youth during global crisis times, such as during a pandemic. Previous research has also found that evidence-based mindfulness interventions were effective for depression and anxiety during the pandemic [91].

Although most of the included studies found improvements in depression and anxiety, not all studies in this review helped with alleviating both anxiety and depression together in a single intervention. However, it is important to stress that it is not unusual for interventions to not work simultaneously for both depression and anxiety given their heterogeneity and the fact that there are fundamental differences in the conditions that may require different therapeutic approaches [92]. A few studies did not find that the interventions reduced both anxiety and depression together [45,61,64,70,80,81]. For example, the 4-week MBSR with MBCT RCT (N=144) by Sun et al [66] found reductions in anxiety but not depression in the intervention relative to the control arms. Furthermore, while the 4-week CBT RCT (N=585) by Rackoff et al [61] found that the intervention reduced depression, there were no significant changes in anxiety. In contrast, the 8-week CBT RCT (N=177) by Simonsson et al [64] found reductions in anxiety but not depression in youth. Both studies had some elements of mindfulness. In addition to this, the 8-week web-based CBT plus mindfulness intervention by Ritvo et al [62] did not have any significant effects on depression or anxiety. This contrasts with a CBT chatbot app that reduced depression and anxiety levels in youth [54].

In addition, deciphering why a minority of the studies did not help for either depression or anxiety is challenging as some studies had common interventional content and duration with others that were effective for the same mental health outcomes. For instance, It could be argued that the 4-week CBT intervention that only found improvements in depression needed a longer duration for it to ameliorate anxiety [66], but a similar study of 4 weeks with a comparable sample size found the opposite [61]. It should be noted that this study combined both MBSR with MBCT, which could theoretically indicate that a shorter study requires a more rigorous combined approach, although more research on this topic is needed. It is also worthwhile to consider the intervention media and specific content. The study that only found improvements in anxiety but not depression integrated mindfulness over WeChat and Zoom (Zoom Video Communications) [66], whereas the study that found the opposite used web-based CBT learning modules [61]. From a technology standpoint, one used video-based communication and chat, whereas the other used a web-based medium with courses. It is interesting to note that the intervention that did not find any improvements in depression or anxiety delivered CBT on the web and also offered peer support. Thus, there is insufficient evidence to be able to discern whether specific interventional content, such as the media through which the interventions were delivered, impacted outcomes.

Our review also found that the effects on stress are consistent with the literature on mindfulness and CBT showing a positive effect on stress [88,93]. The reasons for the lack of consistency in studies during the pandemic are unclear. However, the pandemic was an unprecedented time; hence, the global economic, physical health, and psychological health effects of the pandemic could have impacted the delivery and effectiveness of the interventions. Psychological stress was higher during this time [94]; hence, reducing it to “normal prepandemic times” levels was perhaps more challenging, especially in populations who already had high stress before the pandemic, which further compounded it.

It would be of research interest for future studies to undertake functional magnetic resonance imaging scans to evaluate whether the mindfulness-based interventions lead to actual changes in the brain [95], which could complement subjective self-report measures of changes in mindfulness.

Third, we aimed to determine what KT strategies were used and whether the interventions improved knowledge regarding self-care, mental health, and resilience. We found that there is a gap in the literature when it comes to KT and knowledge dissemination in positive psychology interventions for mental health to youth. Only 7% (3/41) of the studies actually assessed either knowledge (1/41, 2%) or self-efficacy for managing mental health in youth. To understand whether participants actually acquired new knowledge and whether it was disseminated properly, future studies need to measure knowledge levels of positive psychological techniques, including mindfulness, and overall knowledge of mental health care and self-care. It is also important to evaluate self-efficacy for mental health care, and only 4% (2/41) studies examined this. Without understanding whether participants gained knowledge and have perceptions of being capable of taking care of their mental health, the outcomes of these studies are difficult to interpret. Self-efficacy has been previously linked to behavior change [96], highlighting its pertinence to self-care behaviors regarding mental health. Knowledge has been previously found to increase self-help behaviors and awareness of mental health problems in young adults [40]. In addition, by measuring improved knowledge, clinicians can be reassured that knowledge acquisition of new mental health techniques and self-care behaviors assists patients.

Finally, few studies examined knowledge acquired in relation to mental health self-care of mental health literacy. However, it is important to note that a recent systematic review and meta-analysis found that higher levels of mental health self-efficacy specifically do not always lead to behavior change involving help-seeking behavior, and barriers regarding stigma remain [97].

In addition to positive psychology for coping during the pandemic, there are other self-care lifestyle behavioral factors that children and teenagers may modify to improve their mental health. For example, research from the All Our Families cohort in Canada found that improved sleep and reduced screen time were both protective for depression and anxiety in children [98-100]. Research has also found that physical activity is protective of mental health in youth [101]. Thus, interventions and knowledge dissemination efforts may benefit from combined behavioral lifestyle and resilience coping strategies to target child and youth mental health.

### Recommendations and Future Areas of Research and Pandemic Preparedness

We recommend integrating the concept of flourishing, positive psychology, and evidence-based mindfulness programs (eg, MBSR) into existing KT programs. This would involve a unified model of knowledge dissemination and implementation, catered to key stakeholders and mental health first aid training providers. Future studies should also measure knowledge levels of self-care behaviors, including mindfulness or general meditative practice, resilience coping skills, mental health self-care knowledge, knowledge of mental health management, and self-efficacy. Ideally, they should combine resilience-building skills with lifestyle behavioral factors (eg, sleep, physical activity, and screen time modifications). Future studies could consider developing a KT tool that will provide children, teenagers, and their families with useful resources on how to build resilience, meditate, and change their lifestyle behaviors to promote mental health.

Finally, future research should further examine the specific barriers related to implementing these interventions during the pandemic to better prepare for a future pandemic. Nevertheless, it is encouraging to note that most studies successfully implemented their interventions using digital media, something that is more accessible during pandemic-related restrictions and lockdowns. Qualitative research with stakeholders, including the young adult participants, could provide a deeper insight into specific facilitators and barriers experienced during the pandemic, which could help with improving the interventions and future pandemic preparedness.

### Limitations

A limitation of this review is that only 1 reviewer screened the articles due to resource limitations. Ideally, there should be 2 reviewers who screen articles in scoping reviews, reducing bias in selection and in correctly identifying all sources, which makes this more of a narrative literature review. This study is also limited by resources, whereby we accessed free, publicly available articles, which limited the number of databases that were accessed to 3. There is always a possibility that there were more studies that were not included. However, one reviewer redid the search in July 2024 to make sure that no additional studies were missed in the existing databases that were searched.

In addition, we included pretest-posttest 1-arm study designs and did not restrict the inclusion to only RCTs. However, our goal was not to undertake a meta-analysis but to broadly understand what types of interventions were undertaken during the pandemic, considering that randomization was not always possible during the youth mental health crisis throughout the pandemic.

Nevertheless, there are several strengths to this review, including the fact that it comprehensively captured a diverse range of psychological interventions during the pandemic period. There is a possibility that there are more articles that were overlooked given the resource limitations, but this review shows a comprehensive overview of the patterns and types of studies with their common findings during the global COVID-19 pandemic.

### Conclusions

We aimed to review what types of positive psychological interventions or positive psychology–related (with derivatives of positive psychology, such as gratitude, positive emotions, and acceptance) interventions were developed during the global youth mental health crisis of the COVID-19 pandemic to meet the mental health needs of youth during this time. We identified 41 psychological interventions that were undertaken in youth during the pandemic. The studies had mixed findings, but overall, the ones that targeted several positive psychology elements, had clearly defined intervention content, or used evidence-based mindfulness programs had greater effectiveness. The reasons why some mindfulness-based interventions had mixed results during the pandemic are not entirely clear. Finally, there is a gap in the knowledge dissemination and uptake literature, whereby most studies did not assess mental health knowledge; mental health management knowledge; self-efficacy for managing mental health problems; or knowledge levels and self-efficacy for practicing positive psychological techniques, including mindfulness. Future studies should measure knowledge and self-efficacy as part of a KT strategy to promote youth mental health, but they should also consider barriers.

## Appendix

Multimedia Appendix 1 Table 1.

## Abbreviations

CBT: cognitive behavioral therapy
DBT: dialectical behavior therapy
KT: knowledge translation
MBCT: mindfulness-based cognitive therapy
MBSR: mindfulness-based stress reduction
PRISMA-P: Preferred Reporting Items for Systematic Reviews and Meta-Analyses for Protocols
RCT: randomized controlled trial
WHO: World Health Organization
