# Supplementary material for: Psychological Interventions and Those With Elements of Positive Psychology for Child and Youth Mental Health During the COVID-19 Pandemic: Literature Review, Lessons Learned, and Areas for Future Knowledge Dissemination
Source: JMIR Pediatr Parent. 2024 Sep 13;7:e59171. doi: 10.2196/59171 (PMC11437230; doi:10.2196/59171)
Supplement: Multimedia Appendix 1 [file pediatrics_v7i1e59171_app1.pdf]

Table 1. Child and youth positive psychological interventions.

Study	Location; design; duration	Sex and age	MH[a] or psychological measures	BCTs[b], theory, and other targets	Positive psychology intervention type and media	Outcomes	Mindfulness, resilience, and acquired knowledge(KT[c])
Krifa et al [53], 2022	Tunisia (N=366); 2-armed RCT[d]; 8 weeks	94% female; mean age 20.7 (SD 1.6) years	Anxiety, depression (DASS-21[e]), well-being (Warwick-Edinburgh Mental Wellbeing Scale), emotional regulation, hope (State Hope Scale), and optimism	Multiple positive psychological interventions combined	Digital () with lectures, assignments, and videos; positive psychology intervention targeting health care students; founded on compassion, positivity, and meaningful engagement	Significant between-group effects for MH outcomes—depression: *F*=13.6 (DF=1.159), mean score lower by 0.23 points, Cohen *d*=0.28, and *P*<.01; anxiety: *F*=6.82 (DF=1,159), mean score lower by 0.17 points, Cohen *d*=0.21, and *P*<.01; stress: *F*=16.9 (DF=1,159), mean score lower by 0.21 points, Cohen *d*=0.32, and *P*<.01; well-being: *F*=13.4 (DF=1,159) and mean score lower in the control group by −0.27 points;	Knowledge: —[f]; increase in emotional regulation: $F_{1.70\text{-}88}$=37.6; *P*<.01

						hope: *F*=7.33 (DF=1.159), mean score lower in the control group by −0.35 points, Cohen *d*=−0.21, and *P*<.01; optimism: *F*=12.45 (DF=1,159), mean score lower in the control group by −0.23 points, and Cohen *d*=−0.28; emotional regulation: *F*=24.1 (DF=1,159), mean score lower in the control group by−0.50 points, and Cohen *d*=−0.39	
Dupont et al [51], 2022	United States (N=250); RCT; 2 weeks	76.9% female; mean age 18.7 (SD 2.1; range 18-45) years	Positive affect (PANAS[g]) (hedonic wellbeing) life satisfaction (general life satisfaction scale), optimism (Life Orientation Test), loneliness (UCLA[h] Loneliness	Positive psychology; informal mindfulness	Gratitude, acts of kindness, and mindful photography; online	No significant effects of the intervention on outcomes; improvements in positive and negative affect across the intervention and control groups	Knowledge: —[f]

			Scale), perceived social support (Interpersonal Support Evaluation List), perceived stress (PSS[i]), and well-being (Psychological Wellbeing Scale)				
Shabahang et al [63], 2021	Iran (N=150); RCT; 3 weeks	51% female; mean age 24.7 (SD 5.4) years	Anxiety (Short Health Anxiety Inventory and Anxiety Sensitivity Index–3) and COVID-19 anxiety	CBT[j] theoretical model	CBT delivered over video (9 sessions of 15 minutes)	Anxiety decreased in the treatment arm versus control—COVID-19 anxiety: F=139.2 (DF=1); health anxiety: F=42.9 (DF=1); anxiety sensitivity: F=40.4 (DF=1); P<.001	Knowledge: —[f]
Pizarro-Ruiz et al [60], 2021	Spain (N=164); RCT; 2 weeks	Students, 92% female in the intervention arm and 72% female in the control arm; mean age 22.0 (SD 2.6;	Positive and negative affect (PANAS), life satisfaction (Satisfaction with Life Scale), HFS[k], and Brief Strengths Scale	—[f]	Spanish mindfulness app; not much information	Significant between-group differences in satisfaction with life scores (higher in the intervention group); significant between-group differences in facets of resilience (increased in the intervention versus control group; not	Knowledge: —[f]; + increase in mindfulness strength

		range 18-60) years				overall score—temperance strength: *F*=8.86 and *P=0.003*; interpersonal strength: *F*=6.0 and *P=0.015*); forgiveness, including toward oneself(F=5.81)(p=0.017) and others (F-5.65)(p=0.019, increased in the intervention versus control group mindfulness increased (*F*=15.45; *P*<.001)	
Rackoff et al [61], 2022	United States (N=585) ; 4 weeks	71.7% female (intervention group); mean age 20.2 years	Stress and depression	Founded on building purpose, resilience , coping with education , advice/tips, social support and connections, goals, helping with building skills, grief coping mechanisms,	Online CBT with resilience support (modules); control group received standard care	Significant decrease in stress in the intervention group compared with the control group (*d*=−0.18; *P=0.035*); significant decrease in depression in the intervention versus control group (*d*=−0.20; *P=0.018*); no significant between-group differences in anxiety	Knowledge: —[f]

				mindfulness, sleep advice, and self-monitoring/self-awareness			
Dunning et al [50], 2022	United Kingdom (N=460); parallel RCT (psychoeducation control)	Ages between 11-16 years	DERS[m], Child Affective Mindfulness Questionnaire, Warwick-Edinburgh Mental Wellbeing Scale, and Revised Child Anxiety and Depression Scale	Mindfulness; no details on the program	8 sessions of 45 minutes on mindfulness training in person	No significant effects on any outcomes of interest relative to the control group, including mindfulness[l]	Mindfulness knowledge: —; mindfulness did not increase
Bazzano et al [44], 2022	United States (N=86); cluster RCT	46.3% female; age range 11-14 years	Anxiety (SCARED[n]) and depression (PHQA-9[o]); no mindfulness measure	Mindfulness and mind-body movement	In-person mindful breathing exercises and yoga exercises	No statistically significant differences between the intervention and control arms; only effects of time on anxiety	Knowledge: —[f]
Chung et al [46], 2021	Australia and the United Kingdom (N=427); quasi-experimental	84% female; age range 18-44 years	Stress (PSS), well-being (Warwick-Edinburgh Mental Wellbeing Scale), and mindful attention awareness	Mindfulness	Online mindfulness (brief); 6 mindful breathing audios	Perceived stress decreased by 2.8 points on the PSS in the intervention group (R^2=0.08); well-being increased by 0.35 points on the Warwick-	Mindfulness knowledge: —[f]; mindfulness increased

	pilot study; 12 weeks					Edinburgh Mental Wellbeing Scale (R^2=0.35; *P*<0.01); mindfulness increased by 0.22 points (SD 0.09); model associated with a 12% change in mindfulness	
Sun et al [66], 2022	China (N=114); RCT; 4 weeks	73.7% female; mean age 22.1 (SD 2.6) years	Anxiety (GAD-7[p]) and depression (PHQ-9[q])	Founded on MBSR[r] and mindfulness-based cognitive therapy; personal tailoring[l]	mHealth[s] mindfulness targeting resilience versus online social support; Zoom mindfulness classes; audio-based mindfulness over WeChat; learning over videos; group messaging; control received online social support	Significant reductions in anxiety in the mindfulness arm (*d*=0.72; *P=0.024*); reductions in depression in both arms	Knowledge: yes; increase in knowledge of mind and body, self-care, and regulation; learned how to cope with emotions and stress (*P*<.01 relative to control)

Simonsson et al [64], 2021	United Kingdom (N=177); RCT; 8 weeks	64.4% female; age range 18-24 years	Anxiety and depression (PROMIS[t])	CBT and mindfulness; not much detail	Online weekly mindfulness classes (8 in total) founded on a mindfulness book+CBT therapeutic exercises; waitlist control	Significant between-group differences in anxiety—lower levels in mindfulness group (*B*=−0.36; *t*=−2.25; *P=0.025*; no significant effects for depression	Knowledge: —[f]
Ritvo et al [62], 2021	Canada (N=154); 2-arm RCT	76% female; mean age 23.1 (SD 8.0) years	Depression (PHQ-9), anxiety (BAI[u]), stress (PSS), and FFMQ-SF[v]	Building healthy relationships, coping skills, mindfulness, healthy lifestyle (eating), education, and life satisfaction (Brief Multidimensional Students' Life Satisfaction Scale)	Web–based mindfulness CBT (8-week duration, 12 modules); peer support versus a waitlist control	No significant between-group differences on outcomes besides PSS; stress lower in intervention arm (*B*=−2.31; *P=0.03*)	Knowledge of mindfulness: —[f]; nonsignificant difference in the nonjudgment facet scale (*P*=.06)
Gabrielli et al [52], 2021	Italy (N=71)	68% female; mean 20.6	Anxiety and stress (GAD-7 and PSS-10) and	Mindfulness, behavioral coping	Atena chatbot and educatio	Significant reduction in anxiety (t_{39}=0.94;	Knowledge of mindf

		(SD 2.4) years	mindfulness (FFMQ[w])	strategies for emotional regulation, education, advice/tips, and support	nal videos	*P*<=0.009) plus marginal reductions in stress (*P*=.05)[l]; mindfulness facets improved—describing: +1.92 (SD 5.29) points ;nonjudging: +2.17 (SD 6.09) points; *P=0.03*	ulness: —[f]; overall, participants expressed it was enjoyable ; and an opportunity to learn and practice mindfulness; improved nonjudgmental and describing facets
Dorais et al [49], 2021	United States (N=190); RCT; 4 weeks	84% female; mean age 27.1 (SD 7.38) years	Stress (PSS) and CAMRS[x]	Prayer for stress, education, motivation, and reminders	Spiritual meditation, guiding counselor in prayer, videos, and emails	Significant reductions in stress in the intervention arm (*B*=−1.12; SE 0.38; *P<0.01*; 95% CI −1.87 to −0.36); mindfulness increased by 0.94 points (SE 0.33) in the	Mindfulness knowledge: —; overall mindfulness increased

						intervention arm (*P*<.01; 95% CI 0.26-1.62)	
An et al [43], 2022	Vietnam (N=49); RCT; 8 weeks	Mean age 20.0 (SD 0.6) years	Depression, anxiety, and stress (DASS-42[y] and PSS)	Founded on MBSR; foster resilience; EEG[z]	Mindfulness based on 8-week MBSR program	Significant reductions in stress by 33% in the intervention group (mindfulness) versus the control group (*P*=0.002); significant reductions in depression and anxiety (40%) relative to control participants (p-value=0.002; more activity in the brain regions of the frontal and occipital lobe	Knowledge of mindfulness: —[f]; potential proxy for the effects of mindfulness; brain changes on scan; no information on mindfulness levels[l]
Liu et al [54], 2022	China (N=83); RCT; 16 weeks	55.4% female; mean age 23.0 (SD 1.7; range 19-28) years	Depression and anxiety (PHQ-9 and GAD-7); no mindfulness measure	CBT theory founded on emotional regulation, monitoring, education, feedback,	Chatbot based on CBT via WeChat; bibliotherapy control	Significant reductions in depression (*F*=22.8; *P*<.01) and anxiety (*F*=5.3; *P*=0.02)	Knowledge: —[f]

				and support			
Chang et al [45], 2022	United States (N=679); RCT; 8 weeks	71.6% female students; age: —[f]	Stress (PSS), well-being (Warwick-Edinburgh Mental Wellbeing Scale), negative and positive affect (PANAS), resilience (BRS[aa]), and depression and anxiety (brief PHQ-9)	—	Online yoga (Isha Upa) intervention (modules) with guided audios (17 minutes)	Significant reduction in stress among the intervention group versus control (both ITT[ab] and per-protocol analyses): Cohen *d*=0.27 and *P*=0.009; well-being differences between arms: *d*=0.32 and *P*=0.002; lower depression in intervention arm[ac] (week 2 only): *d*=0.18 increase in positive affect (weeks 2-4): *d*=0.28; all other outcomes: nonsignificant differences between groups, including resilience × group over time	Knowledge: —[f]; no change in resilience
Colaianne et al [47], 2022	United States (N=601); longitudinal quasi-experimental	52%	Self-Compassion Scale–Short Form, Multidimensional Assessment of Interoceptiv	Self-care, meditation, and compassion skills	Elements of mindfulness; web-based , and face-to-face	Moderate effects of the intervention on self-compassion (*g*=0.41, 95% CI 0.18-0.68); interoceptive awareness increased	Knowledge: not directly assessed but elements

	study; September, January, and April data collection (7 months)		e Awareness			(*g*=0.73, 95% CI 0.36-1.01)	related to behavior change and positive learning attitudes including: —[f]; motivation to practice compassion and continue learning: 8.8/10 (SD 2.06)
Cruwys et al [48], 2021	Australia (N=174); RCT; 1 year	76.8% female in the intervention group and 73.3% female in the control group; mean age	UCLA Loneliness Scale, well-being (Short Warwick-Edinburgh Mental Wellbeing Scale), and depression (DASS-21)	Provision of social support; social identity framework	CBT social support (5 sessions over 8 weeks) for both intervention arms	A social support intervention is superior to CBT for mental health during the pandemic; significant effects of group social support on depression (χ^2=31.4; (DF=16) *P*<.001), loneliness (χ^2=21.6; DF=8	Knowledge: —[f]

		18.9 (SD 1.9) years				P=0.06), and well-being (χ^2=23.0; DF=8; P=0.003)	
Suffoletto et al [65], 2021	United States (N=52); pilot RCT; 12 weeks	79% female in the intervention group and 100% female in the control group; mean age 18.7 years	Depression, mental health self-efficacy (MHSES[ae]), and College Counseling Center Assessment of Psychological Symptoms	CBT and DBT[af]+psychology principles, social support, and emotional regulation	MoST-MH[ag] intervention, digital (mHealth) web–based social support ("check ins"), SMS text messaging, education/awareness, advice/tips, feedback, support, and videos on MH	Significant reductions in depression in the intervention versus control group (d=0.36, 95% CI 0.08-0.64); no significant changes in mental health self-efficacy[l]	Knowledge: —[f]; MH self-efficacy: no changes; self-management self-efficacy low: 91%
Ludin et al [55], 2022	New Zealand (N=127); feasibility study with qualitative interviews	Ages of 13-24 years	COVID-19 anxiety	CBT+positive psychology	Aroha Chatbot app; CBT+positive psychology; messages via Facebook Messenger	Decrease in COVID-19 anxiety by 0.8 points (SD 0.1); users found the chatbot to be helpful and acceptable	Knowledge: —[f]

Miller et al [57], 2021	United States (n=35 teenagers and n=32 parents); RCT 12 weeks	37% female teenagers; mean age of teenagers 12.9 years	PTSD[ah] (Child PTSD Symptom Scale), Brief Problem Monitor, and FFMQ	Mindfulness+mentoring; social support	Mindfulness and mentoring	Teenagers assigned to mindfulness mentoring had an increase in the facet of mindful describing (*B*=1.65; SE 0.39; p-value<0.001 Group by time interaction effects for lower PTSD in the mindfulness plus mentoring group p-value=0.002	Knowledge: —[f]
Tymofiyeva et al [67], 2022	United States (N=21); pilot intervention study; 12 weeks	50% female in the intervention group and 55% female in the control group; ages of 14-18 years	Emotional well-being (SDQ[ai]) and Child Outcome Rating Scale; no mindfulness measure	Mindfulness	Digital mindfulness intervention (TARA[aj]) over Zoom (during the pandemic; 7 sessions)+face-to-face (before the pandemic; 5 sessions)	No significant effects of the intervention on emotional well-being compared with the control (only in exploratory analysis)	Knowledge: —[f]
Yadav et al [68], 2021	United States (N=40); pretest-posttest experi	89% female; ages of 14-18 years	Stress (PSS) and depression (PHQ-9); no mindfulness measure	Heartfulness meditation+brain wave entrainm	Group video calls 30-minute mindful breathin	No effects on the audio brain wave group; the mindfulness group experienced	Knowledge: —[f]

	mental study randomized to 4 groups; 4 weeks			ent together and separately compared with a no-intervention control; social support/peer support; education	g sessions versus audio group that listened to specific-frequency sounds (BrainTap app)	reductions in stress (t=3.2) (P=value=0.008) and depression (t=2.7; P=0.003)	
Yuan [69], 2021	China (N=84 in the intervention) (N=90 control) RCT	51% female; age range of 12-14 years	Resilience (Connor-Davidson Resilience Scale)	Mindfulness	Mindfulness	Increased levels of resilience in the mindfulness group over time (F=734.3; P<.001; note that there was a higher resilience at baseline in the control group)	Knowledge: —[f]
Orengo-Aguayo et al [59], 2022	United States in collaboration with Puerto Rico (N=56); pretest-posttest 1-arm intervention study	Ages of 5-18 years	Spanish version of the Child PTSD Symptom Scale and Spanish version of the revised Depression and Anxiety Scale	Trauma-focused CBT training; psychologists were trained to deliver CBT (15)	Digital CBT telehealth+face-to-face	Significant reduction in PTSD (Cohen d=1.32), depression (d=1.32), and anxiety (d=1.18) in participants; training of psychologists was successful	Knowledge: —[f]

Nicol et al [58], 2022	United States (N=37); pilot RCT; 12 weeks	88% female; ages of 13-17 years	Depression (PHQ-9), anxiety (GAD-7), Mental Health [58] Scale Confidence in Managing Mental Health Issues Scale	CBT	Digital chatbot app; CBT	Mean PHQ-9 scores decreased by 3.3 points in the intervention versus control group (2 units); mean GAD-7 score was 4.3 points lower in the intervention than the control group; mental health self-efficacy increased by 0.7 points relative to the control group (95% CI −3.4 to 17.3; *d*=0.71)	Knowledge: —[f]; higher mental health self-efficacy
Malboeuf-Hurtubise et al, 2021	Canada (N=37); cluster-randomized trial	43% female; mean age 8.1 years	Basic psychological need satisfaction and mental health difficulties (Behavior Assessment System for Children); no mindfulness measure	Mindfulness-based I[ak] (body scan, mindful walking, gratitude, and journaling) versus P4C[al]	Group–based telehealth+psychology mindfulness+P4C	Effect of mindfulness on on mental health challenges ($p=0.016$) $F=5.58$ (DF= 1,29) Mindfulness group had higher psychological need satisfaction levels (mean score 1.21 points higher versus 0.35 points lower in the P4C group); the P4C group had better scores on mental health than the mindfulness group (1.34 points lower in	Knowledge: —[f]

						the P4C group versus 0.7 points higher in the mindfulness group	
Dai et al [70], 2022	China (N=120); RCT; 6 weeks	76.8% female in the intervention group and 82.1% female in the control group; mean age 19 years	DASS-21 and Chinese short FFMQ	General mindfulness-based intervention; audio+video instructions and homework	Online mindfulness intervention (WeChat)	Anxiety (p-value<0.001), stress (p=0.003), and mindfulness (p=0.005) improved; no improvements in depression	—[f]
Dwidiyanti et al [71], 2021	Indonesia (N=70); quasi-experimental pretest-posttest study; 1 month (May-June)	Percentage of female participants: —[f]; age range 18-22 years	Beck Depression Inventory–II	DAHAGA[am] Islamic mindfulness-based intervention centered on spirituality	Android Islamic mindfulness spiritual app	Significant within-group (−5.71, SD 0.45 points) and between-group improvements in depression (*P*<.001)	—[f]
González-García et al [72], 2021	Spain (N=66); pretest-posttest design; 16 days	—[f]	Stress (PSS) and anxiety (STAI[an])	Centered on compassion and mindfulness	Brief online mindfulness intervention; online platform	Reductions in anxiety (*g*=0.5146) and stress (*g*=0.606; *P*<.001); improved self-compassion (*g*=0.69; *P*<.001)	—[f]

Guzick et al [73], 2022	United States (N=129 parents); retrospective analysis of a pretest-posttest study questionnaire; 6 weekly sessions	50% female; age range 5-13 years (children)	Spence Children's Anxiety Scale and PROMIS Parent Proxy short form	Centered on cognitive behavioral principles	CBT; parents were leaders; 6 sessions over video	Assisted with anxiety (*d*=0.56), stress (*d*=0.61), and depression (*d*=0.69; *P*<.001)	—[f]
Hanani et al [74], 2022	Palestine (N=329; second phase N=66; RCT; 8 weeks	Percentage of female participants: —[f]; mean age 19.5 (range 17-27) years	12-item GHQ[ao]	General CBT principles	CBT sessions	Improvement in depression+anxiety (2.7-point reduction in the intervention , with significant between group differences group p=0.01	—[f]
Karampas et al [76], 2022	Greece (N=26); RCT; 5 weeks	96.2% female; mean age 24.46 years	DASS	General acceptance and stress mindset	Stress mindset program and acceptance and commitment therapy; ReStress Mindset CBT online; 5 weekly	Improved self-efficacy for stress and led to reductions in a mindset that is influenced by stress (score of 1.19 vs 2.27 in the control group)	—[f]

					sessions online		
Harper et al (2022)	USA N=8 Pilot study (pre to post) 4 weeks	F=50% Ages 11-15	Patient Health Questionnaire-9 Generalized Anxiety Disorder	Mental health Anxiety	Web-based sessions Telehealth app CBT	No stat significant reductions in anxiety	—[f]
Malboeuf-Hurtubise et al, 2021	Canada (N=22); pilot cluster RCT; 5 weeks	50% female; mean age 11.3 years	Mindful Attention Awareness Scale and Behavior Assessment System for Children	Art for emotional regulation+focus	Digital online art therapy involving emotional directed art (2 groups and mandala drawings)	No significant effects of the intervention on depression, anxiety, or mindfulness	—[ae]
Midgley et al [80], 2021	United Kingdom (N=23); 1-arm pilot study; 10 weeks	78% female; age range 16-18 years	Depression (Quick Inventory of Depressive Symptomatology, Adolescent version) and anxiety (GAD-7)	Targeted affect	Online psychodynamic therapy internet based focused on affect therapy	Reductions in depression by 4.43 points (*P*=0.0028); no significant effects on anxiety	—[f]
Sturgill et al [82], 2021	United States (N=99); over a 14-week term	69% female; age range of 18-29 years	Anxiety (GAD-7) and depression (PHQ-9)	Mindfulness and emotional intelligence	Mindfulness app that uses artificial intelligence	Reductions in depression (4 points; p=0.001) and anxiety (5.2 points; *P*<0.001.)	—[ae]

					(Ajivar chatbot)		
Zúñiga et al [83], 2021	Chile (N=123); pretest-posttest observational study; 1 month	48.8% female; fourth-year medical students	Stress (PSS), Maslach Burnout Inventory, and Connor-Davidson Resilience Scale	Use of self-care and mindfulness	Self-care mindfulness-based course	2-fold improvement in mindfulness relative to baseline (25%); reduction in stress burnout by half; improved resilience between 2 time points (*P*<.001)	—[f]
Klim-Conforti et al [77], 2021	Canada (N=530); RCT	Age range 11-14 years	Revised Child Anxiety and Depression Scale	Harry Potter CBT skills	CBT delivered by teachers	The intervention group had better scores for depression and anxiety relative to the control group (*t*=2.96; *P*=.001)	—[f]
Liang et al [78], 2021	China (N=52); randomized intervention; 4 weeks	62% female; mean age 20.7 years	PHQ-9 and GAD-7	DBT mindfulness and emotional skills	DBT	Significant improvement in depression (*t*=2.5; *P*=.014) and anxiety (*t*=3.7; *P*≤.001)	—[ae]
Mirabito and Verhaeghen [81], 2022	United States (N=111); RCT; 4 weeks	—[ae]	Depression and anxiety (DASS-21) and mindfulness (FFMQ)	Mindfulness skills	Online Koru mindfulness group based	Improvements in stress (*d*=0.65; *P*<.001) and anxiety (*d*=0.37; *P*=0.021) but not depression	—[ae]

[a]MH: mental health.
[b]BCT: behavior change technique.
[c]KT: knowledge translation.
[d]RCT: randomized controlled trial.
[e]DASS-21: Depression, Anxiety, and Stress Scale–21.
[f]Not applicable.
[g]PANAS: Positive and Negative Affect Schedule.

[h]UCLA: University of California, Los Angeles.
[i]PSS: Perceived Stress Scale.
[j]CBT: cognitive behavioral therapy.
[k]HFS: Heartland Forgiveness Scale.
[l:] annotation for a specific group
[m]DERS: Difficulties in Emotion Regulation Questionnaire.
[n]SCARED: Screen for Child Anxiety Related Emotional Disorders.
[o]PHQA-9: Patient Health Questionnaire–9 modified for Adolescents.
[p]GAD-7: Generalized Anxiety Disorder–7.
[q]PHQ-9: Patient Health Questionnaire–9.
[r]MBSR: mindfulness-based stress reduction.
[s]mHealth: mobile health.
[t]PROMIS: Patient-Reported Outcome Measurement Information System.
[u]BAI: Beck Anxiety Inventory
[v]FFMQ-SF: Five Facet Mindfulness Questionnaire–Short Form.
[w]FFMQ: Five Facet Mindfulness Questionnaire.
[x]CAMRS: Cognitive and Affective Mindfulness Revised Scale
[y]DASS-42: Depression, Anxiety, and Stress Scale–42.
[z]EEG: electroencephalogram.
[aa]BRS: Brief Resilience Scale.
[ab]ITT: intention to treat.
ac-
ad- not available
[ae]MHSES: Mental Health Self-Efficacy Scale.
[af]DBT: dialectical behavior therapy.
[ag]MoST-MH: Mobile Support Tool for Mental Health
[ah]PTSD: posttraumatic stress disorder.
[ai]SDQ: Strengths and Difficulties Questionnaire.
[aj]TARA: Training for Awareness, Resilience, and Action.
[ak]MBSI:
[al]P4C: Philosophy for Children.
[am]DAHAGA:
[an]STAI: State-Trait Anxiety Inventory.
[ao]GHQ: General Health Questionnaire.
